# Seroprevalence of Specific Antibodies against SARS-CoV-2 from Hotspot Communities in the Dominican Republic

**DOI:** 10.4269/ajtmh.20-0907

**Published:** 2020-10-21

**Authors:** Robert Paulino-Ramirez, Amado Alejandro Báez, Alejandro Vallejo Degaudenzi, Leandro Tapia

**Affiliations:** 1 Instituto de Medicina Tropical & Salud Global, Universidad Iberoamericana, Santo Domingo, Dominican Republic;; 2 COVID-19 Emergency Presidential Committee, Santo Domingo, Dominican Republic;; 3 Department of Emergency Medicine, Medical College of Georgia, Augusta, Georgia;; 4 Center of Operational Medicine, Universidad Nacional Pedro Henriquez Ureña, Santo Domingo, Dominican Republic

## Abstract

Seroprevalence surveys are of utmost importance to assess the proportion of a population that has developed antibodies against a newly introduced virus and could therefore potentially exhibit immunologic protection against subsequent infection. This study aims to understand the distribution of IgM and IgG antibodies in the Dominican Republic. We surveyed a total of 12,897 participants between April and June 2020 in 10 provinces of the Dominican Republic. Survey efforts in emerging hotspots yielded a positivity for all participants of anti–SARS-CoV-2 IgM of 3.8% and IgG of 5.4%, indicating that the pathogen was in circulation before the identification of those particular communities as hotspots. We found important age differences between participants who participated in the serological study where a higher mean age is associated IgM positivity and a lower age with IgG positivity. Our results highlight the need for strategies that involve community-based seroprevalence monitoring. These should preclude syndromic case identification. Also, the higher mean age of IgM-positive participants suggests that strategies based on syndromic surveillance could identify hotspots at later phases, based on the number of cases detected at the healthcare center, as such community-based seroprevalence monitoring may be an effective intervention for future outbreaks.

The Dominican Republic reported its first confirmed case of SARS-CoV-2 on March 1, 2020.^1^ As of July 23, the country had reported 57,615 confirmed cases, with 29,704 of those still active cases and a total of 1,006 deaths.^2^ The Dominican Republic’s health response against this pathogen consisted of strict limitations of commercial activities, suspension of in person instruction in schools and universities, and nighttime curfews starting on March 19. Decisions to loosen restrictions were made based on declining positivity rates and increases in hospital capacity to admit COVID-19 cases.^1,2^

Because of a sudden increased demand for confirmatory diagnostic testing, mildly affected and asymptomatic individuals have limited access to laboratory testing. As a result of such circumstances, the number of confirmed SARS-CoV-2 infections can significantly underestimate the actual number of cases.^3^ Besides this, the known differences in the proportion of asymptomatic and symptomatic manifestation by varying age-groups can lead to under detection in younger populations and overestimation of severity in older communities in countries with only syndromic surveillance.^4–6^ To expand testing capacities during initial phases of the pandemic caused by SARS-CoV-2, rapid detection of antibodies by the ELISA method was implemented as a screening method for active and passive surveillance. This method was mainly based on detecting specific antibodies against SARS-CoV-2 antigens, where IgM antibodies are the first antibodies that are created in response to initial exposure to an antigen and IgG antibodies appear at a later phase and serve as immunologic memory.

As seen in many countries, changing testing strategies during epidemics makes it nearly impossible to estimate the extent of the population exposed to the pathogen at a given moment. However, this information is crucial in planning evidence-based strategies for lifting physical distance and confinement measures. In this context, seroprevalence surveys are of utmost importance to assess the proportion of the population that has already developed antibodies against the virus and could potentially exhibit immunologic protection against subsequent infection.^7^ As recommended by the WHO, monitoring seroprevalence changes over time is crucial to anticipate the epidemic’s dynamics and plan an adequate public health response to contain the spread of the pathogen or prevent its reemergence.^4^ In addition to this, seroprevalence studies offer the benefit of saving testing costs and time and the possibility of carrying out community-based intervention in identified emerging hotspots to stop further spread of the disease.^8^

This study aimed to understand the distribution of IgM and IgG antibodies within the Dominican Republic during community-based interventions. To achieve this, we analyzed the demographic characteristics of participants who received a SARS-CoV-2 IgM/IgG rapid test in emerging hotspots within the Dominican Republic. Emerging hotspots were considered as an increased rate of new infections compared with the previous epidemiological week in a given municipality or province. Because of the inherent difference in IgM and IgG function and structure, we consider that intervened communities with an increased proportion of IgM antibody positivity indicate an early identification of community circulation of SARS-CoV-2. By contrast, a high IgG combined with a low IgM positivity proportion would suggest a late community intervention. We also consider that differences in antibody profile distribution within age-groups can help identify crucial pockets of transmission within the population and guide public health policies.

To assess the seroprevalence of anti–SARS-CoV-2 antibodies in communities within the Dominican Republic, we contacted individuals randomly in communities identified as emerging hotspots for SARS-CoV-2 by Ministry of Health reports and invited members of households to participate in the seroprevalence study. We collected demographic data for each participant and evaluated anti–SARS-CoV-2 IgM/IgG antibodies using a commercially available chromatographic test targeting the S1 domain of the SARS-CoV-2 spike protein (GenBody Inc., Seoul, Korea). To establish the IgG/IgM concentration, sample, strips were subjected to a colorimetric assay using an automatic analyzer (Food and Drug Administration [FDA] evaluation report: IgM sensitivity (40.0%)/specificity (98.8%), versus IgG sensitivity (56.7%)/specificity (100%) Confiscope G20, Genbody Inc.).^9^ Testing efforts consisted of consecutive weekly serological interventions between April and June in communities identified as emerging hotspots for SARS-CoV-2 infections by the syndromic surveillance program of the country’s national surveillance system. We adhered to ethical guidelines by performing each test under strict confidentiality.

We surveyed a total of 12,897 participants between April and June in 10 provinces of the Dominican Republic. The sampled population consisted of 51% (*n* = 6,597) males with a mean age of 42 years (Table 1). The month of May had the highest turnover of participants, consisting of 53.5% of all the surveyed participants. In part, it can be explained by the Ministry of Health’s enhanced efforts through its multi-sectoral intensive plan to increase testing capacity and early detection and intervention at community and hospital levels.^10^

**Table 1:** Descriptive statistics for seroprevalence efforts in identified emerging hotspots for SARS-CoV-2 in the Dominican Republic (*N* = 12,897)

Demographic characteristics	*N*	%
Gender		
Male	6,597	51.2
Female	6,300	48.9
Age (years)		
Mean age: 42
0–17	379	2.9
18–34	4,399	34.1
35–54	5,380	41.7
> 55	2,739	21.2
Provinces		
Distrito Nacional	2,776	21.5%
Duarte	825	6.4%
La Altagracia	995	7.7%
La Romana	261	2%
La Vega	442	3.4%
Puerto Plata	63	0.5%
San Cristobal	1,616	12.5%
San Jose de Ocoa	113	0.9%
Santiago	1,129	8.8%
Santo Domingo	4,677	36.3%
Sampling dates		
April	1,314	10.2%
May	6,861	53.5%
June	4,660	36.3%
Antibody results		
Positive IgM	491	3.8%
Negative IgM	12,406	96.2%
Positive IgG	704	5.5%
Negative IgG	12,193	94.5%

The provinces of Santo Domingo and Distrito Nacional, which combined make up the Great Santo Domingo Metropolitan Area, accounted for 57.8% of the surveyed population (Figure 1). The third most impacted province was San Cristobal. This province is adjacent to the Great Santo Domingo and is tied to this area by joint economic practices, making these metropolitan areas the most critical hotspots (Table 2). Population dynamics between these economically active and dependent provinces were not studied, but the implications of sustained transmission between these provinces cannot be fully elucidated.^11^

**Figure 1.**
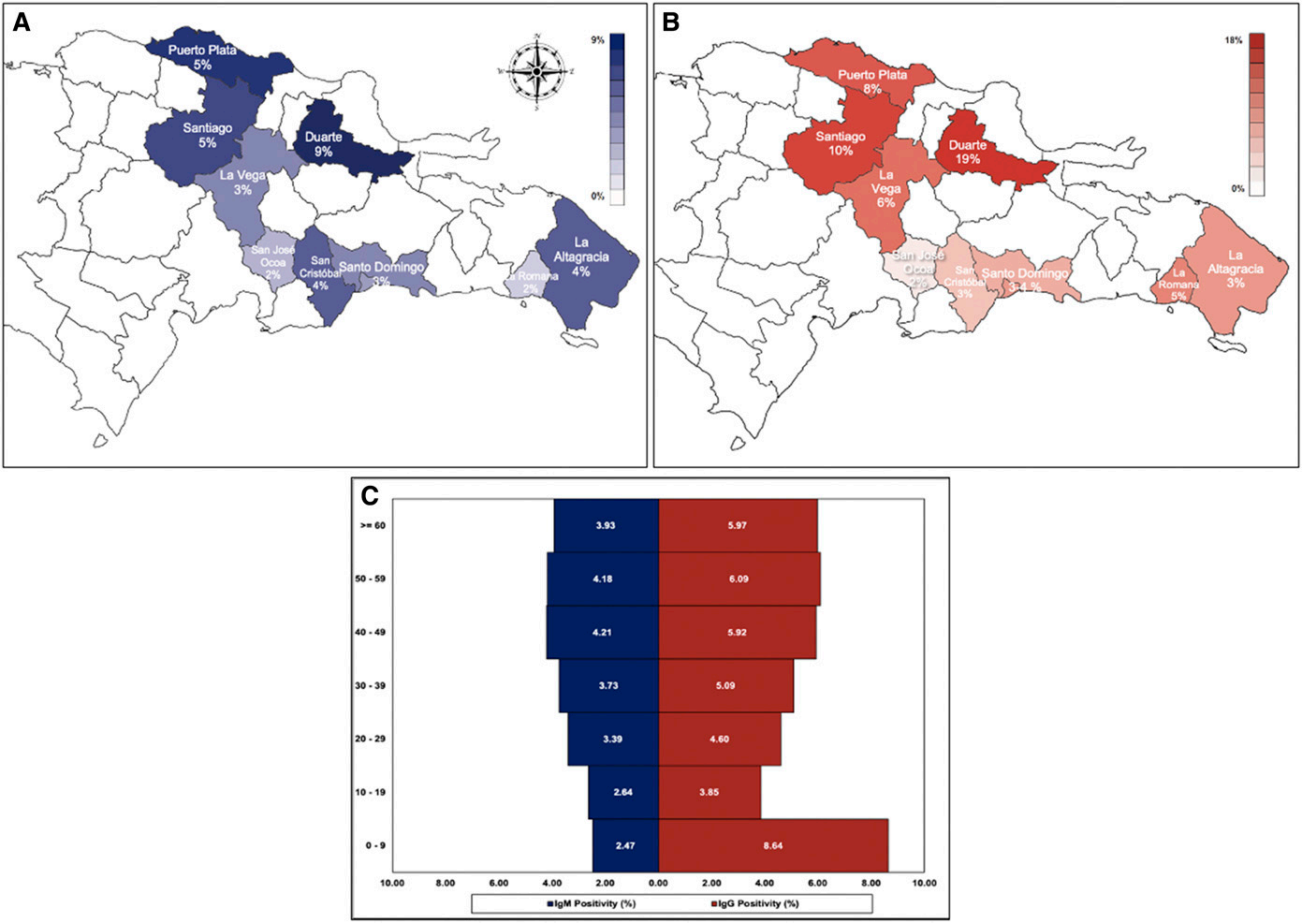
(**A**) Frequency of SARS-CoV-2 IgM antibodies and (**B**) frequency of SARS-CoV-2 IgG antibodies. (**C**) Age distribution and positive IgG/IgM ratio in selected communities in the Dominican Republic.

**Table 2:** Distribution of positive antibody identification linked to emerging hotspots for SARS-CoV-2 in the Dominican Republic (*N* = 1,025)

	IgM, *n* (%) (491 [3.8])	IgG, *n* (%) (703 [5.5])
Distrito Nacional (8,174)*	88 (3.2)	123 (4.5)
May	69 (4.2)	99 (6.1)
June	19 (1.7)	24 (2.2)
Duarte (876)	76 (9.2)	153 (18.6)
April	65 (11.6)	134 (23.8)
May	11 (4.2)	19 (7.2)
La Altagracia (788)	35 (3.5)	38 (3.8)
May	26 (4.1)	23 (3.6)
June	9 (2.5)	15 (4.2)
La Romana (834)	4 (1.5)	14 (5.4)
May	4 (1.5)	14 (5.4)
La Vega (1,297)	15 (3.4)	26 (5.9)
May	15 (3.4)	26 (6.7)
Puerto Plata (479)	3 (4.8)	5 (7.9)
May	3 (4.8)	5 (7.9)
San Cristobal (1863)	57 (3.5)	54 (3.4)
May	15 (2.7)	21 (3.7)
June	42 (4)	33 (3.1)
San Jose de Ocoa (67)	2 (1.8)	2 (1.8)
June	2 (1.8)	2 (1.8)
Santiago (2,249)	53 (4.7)	114 (10.1)
April	46 (6.1)	95 (12.6)
May	7 (1.9)	19 (5)
Santo Domingo (9,448)	158 (3.4)	174 (3.7)
May	102 (3.8)	111 (4.1)
June	56 (2.8)	63 (3.2)

* Selected communities and confirmed (real time polymerase chain reaction) cumulative cases by June 30, 2020 = 26,137.

Survey of all participants in emerging hotspots yielded positivity of anti–SARS-CoV-2 IgM of 3.8% and of anti–SARS-CoV-2 IgG of 5.5%, indicating pathogen circulation before the identification of the community hotspot (Figure 1). Significant proportional differences in antibody positivity between IgM and IgG tests (*P* = 2.8 E-10) highlight the weakness of a reactionary response or symptomatic surveillance with this pathogen where time is essential. By contrast, IgM detection in asymptomatic participants by community intervention is an important component of the efforts to stop the community spread of SARS-CoV-2. This is shown by a decline in IgM positivity in continuous months of implementation. Novel approaches to detect emerging hotspots, such as viral detection in sewage wastewaters, have to be adopted to ensure a faster and earlier response to this pathogen.^12^

We found important age differences between those who participated in the serological study. The 491 surveyed participants who received a positive IgM result (*M* = 43.1, SD = 14.5), compared with the 12,406 participants who tested negative (*M* = 41.9, SD = 16.2), demonstrated significantly higher age at the time of the serosurvey (*t* [533] = −1.6, *P* = 0.05). In addition, the 704 surveyed participants who received a positive IgG result *M* = 41.9, SD = 16.2), compared with the 12,406 participants who tested negative (*M* = 43.1, SD = 16.4), demonstrated significantly lower age at the time of the serosurvey (*t* [12,895] = −1.9, *P* = 0.02). Both of these findings highlight the possibility of intense pathogen circulation before the intervention of an identified hotspot, with IgG suggesting increased immune memory in younger participants, which would lead to decreased susceptible populations, hence a decreased IgM positivity.

Because of substantial differences in severity, complications, and mortality across age-groups, interventions focused on stopping the spread of SARS-CoV-2 should consider different surveillance strategies to reflect such variations.^4^ We found a lower proportion of IgM positivity, 2.1%, in children, than the 3.8% mean, and a higher IgG positivity of 5.8% versus the 5.5% mean, which might indicate cryptic transmission at community levels, especially with this age-group. This scenario has also been explored in other parts of the world, where studies found that children functioned as principal transmission foci because of a high asymptomatic proportion of infections.^13–15^ With the intense debate about whether to reopen schools and colleges in the fall, it should be a public health priority to understand the role of SARS-CoV-2 transmission in seemingly unaffected population and their role in the reemergence of the pathogen.

Strategies involving community-based seroprevalence monitoring should preclude syndromic case identification.^1,16^ Because our study follows seroprevalence intervention in already identified hotspots by symptomatic surveillance and contact tracing, we can assume that the differences in antibody proportions in young populations suggest important pathogen circulation before community detection and intervention. We consider that an essential limitation to our study is the lack of pre-intervention serodata, and assay-related specificity/sensitivity; this might limit our understanding of any specific age-groups that may act as transmission pockets of the SARS-CoV-2 pathogen before the emergence of the hotspot. To counteract this limitation, we base our conclusions on prior viral circulation based on IgG positivity. By doing so, our hypothesis is supported by our findings that show a younger mean age of IgG-positive individuals than negative IgG participants. Also, the higher mean age of IgM-positive participants contributes to our suggestion that strategies based on syndromic surveillance could identify a hotspot at a later phase, based on the number of cases detected at the healthcare center.
